# Comparative palatability of five supplements designed for cats suffering from chronic renal disease

**DOI:** 10.1186/2046-0481-67-10

**Published:** 2014-05-19

**Authors:** Natalia Bernachon, Sandrine Fournel, Hugues Gatto, Patricia Monginoux, David McGahie

**Affiliations:** 1 Medical Department Virbac, 13ème Rue, 06511 Carros, France; 2 R&D Virbac, Carros, France; 3 UPL Virbac, Carros, France

**Keywords:** Renal, Phosphate binder, Cat, Chronic kidney disease, Uraemic toxin binder, Palatability, Compliance, Supplement, Prehension, Consumption

## Abstract

**Background:**

Intestinal phosphate binders, uremic toxin binders and some other types of supplements are an integral part of the management of chronic kidney disease (CKD) in various species, including cats. This pathology in domestic carnivores requires life-long nutritional and medical management. In this context, the compliance of owners and patients cannot be achieved without an adequate level of palatability for oral medication or supplementation. Knowing that hyporexia and anorexia are among the most commonly seen clinical signs in cats suffering from CKD this is already, in itself, a serious obstacle to acceptable compliance in sick animals. The aim of the present study was to investigate the palatability of four commercially available products designed for cats suffering from CKD: Ipakitine® (Vetoquinol, France), Azodyl® (Vetoquinol, USA), Renalzin® (Bayer, France), Rubenal® (Vetoquinol, France) and an additional recently developed product: Pronefra® (Virbac, France). The study was performed with a group of previously-characterised cats, all living in an enriched and well-being securing environment of an independent centre housing panels of pets expert in palatability measurement. In total 172 monadic testings were performed. The palatability of each product was assessed by measuring their rates of prehension and consumption, and the consumption proportions were also analysed.

**Results:**

The most palatable presentation (based on useful consumption) was Pronefra®, which was significantly higher than Azodyl® (p = 0.046), Ipakitine® (p < 0.0001), Renalzin® (p < 0.0001) and Rubenal® (p < 0.0001). The product with the highest rate of prehension was also Pronefra®, which was significantly higher than Azodyl® (p = 0.0019), Ipakitine® (p = 0.0023), Renalzin® (p = 0.0008) and Rubenal® (p < 0.0001).

**Conclusion:**

Pronefra® was the most palatable presentation tested, meaning it may be useful for improving ease of supplementation in CKD cats.

## Background

Chronic kidney disease (CKD) is one of the most common pathological conditions seen in domestic carnivores, especially elderly cats. This disease is known to affect more than 30% of cats over 10 years of age [1], and the mean age for diagnosis is reported to be close to 13 years [2]. It has been recognised that nutritional intervention plays a vital role in the management of many diseases in aging cats and has a special role in the management of renal disorders [3]. Currently, several oral supplements are marketed with proposed benefits for multiple aspects of renal disease including intestinal phosphate binders (IPB), uremic toxin binders, agents intended to reduce progression of fibrotic changes, antioxidants and vitamin supplements. Unfortunately, these products are intended for use in a group of animals already well-known to have poor compliance [4] and none of the existing products appear to respond to the practical need to have one supplement associating several aspects of nutritional management in a highly palatable and easy-to-give form.

Three main targets can be seen in current nutritional management of CKD cats: restriction of available dietary phosphorus, reducing absorption of uremic toxins and reduction of the progression of fibrotic changes. Management of uremic toxins has a direct and practical interest for the CKD patient because anorexia, hyporexia, nausea and vomiting are common and prominent signs of uremia [5]. The benefits of simultaneous use of chitosan and phosphate binders to reduce intestinal ammonia absorption in cats have been shown previously [6] and are an accepted part of the dietary management of CKD in the dog [7].

Glomerulosclerosis, tubulointerstitial lesions, inflammation and fibrosis are common findings in CKD and seem to be independent from the initiating factor of the disease [5]. Some natural agents have shown encouraging results and appear to be efficient in reducing renal inflammation and fibrosis in laboratory animals and humans [8,9], thus justifying use of such supplements in domestic carnivores.

However, of all recommended management options for CKD the current consensus is that restriction of available dietary phosphorus is the major contributor in slowing the disease progression and improving survival times [6,10-14]. The regulation of serum phosphorus concentrations in the body is dependent on the balance between dietary intake of phosphorus and its excretion rate [15]. The kidneys have a pivotal role in the regulation of phosphorus levels because they are the first and the main route of phosphorus excretion in cats [5]. Even if dietary intake remains stable, the decline in the glomerular filtration rate (GFR) in animals with CKD will be translated into hyperphosphatemia and result in an activation of compensatory mechanisms such as secondary hyperparathyroidism which are often deleterious for the kidney itself. Secondary hyperparathyroidism can be seen even in the early stages of CKD in domestic carnivores where ionized calcium and serum phosphorus concentrations often remain within normal ranges [5,10,16,17]. Probably these “normal-range” phosphorus levels are slightly higher than physiologically normal for the individual cat (despite being within the laboratory reference ranges) and/or are maintained only through constantly high levels of PTH [12].

Thus management of the dietary availability of phosphorus in patients suffering from chronic renal disease and/or hyperparathyroidism could be useful from the early stages [10]. Where possible this should be based on accurate monitoring, tailored to the individual cat [12].

The majority of dietary phosphorus comes from protein-rich ingredients in the diet [12,18,19]. Three options are currently available for the management of dietary phosphorus availability in CKD patients [19]: change to a diet specially restricted in phosphorus and/or in protein, use of IPB or both of these in combination. However, the loss of lean body mass is one of the factors associated with progressive CKD in cats [13] and is due to impaired absorption of nutrients from the digestive tract, reduced appetite, nausea, vomiting and malnutrition related to disease progression. It has been reported to be associated with shorter survival [2] and even to be predictive of clinical deterioration in CKD patients [20]. Beyond the scope of CKD, a large proportion of elderly cats, more than 9 years of age, have a tendency to lose body weight due to impaired digestion of proteins [3]. Thus in most cases maintenance of the dietary protein intake (and the proportion of energy obtained from dietary protein) may be required to help maintain lean body mass (LBM) in aging cats [3]. It has been reported that the addition of IPB to a diet with a normal protein content is often sufficient to control phosphate levels in cats in IRIS stage 2 CKD [10] Thus, phosphate restriction with simultaneous maintenance of the standard protein level seems to be preferable in the early stages of CKD [19]. Progressive loss of the functional ability of the kidneys will progressively require a shift to a low phosphorus renal diet and, if necessary, also IPB in the later stages of the disease [10].

Despite the fact that there is scientific evidence for the benefits of IPB [10,12,15,21] a recent survey in the United States performed in 1080 cats has shown that the majority of cats (78,8%) with CKD are not receiving a phosphorus binding agent [22]. Market research studies in Europe show that IPB are prescribed only in 41% of CKD patients (in both dogs and cats) [23]. Practitioners report a lack of palatability of some supplements [5] and the galenic forms are not always well adapted to cats resulting in poor compliance. This problem is even more in evidence when there is a need to provide multiple products to manage multiple aspects of the disease. Palatability has been cited as one of the major factors that influence the selection of a phosphate-binding agent given the fact that in cats presented with azotemia their appetite is often variable or may be selective for certain foods [5]. For this reason the palatability of products designed specifically for CKD cats should be of primary importance in order to avoid inappetence due to the nature of the product.

The goal of this study was to assess, in a panel of standardised cats, the comparative palatability of the available supplements for cats with CKD along with an additional supplement that was in the late stages of development at the time.

## Methods

### Animal selection and test procedure

The study was done with a group of previously-characterised cats, all living in the enriched and well-being securing environment of an independent centre housing panels of pets expert in palatability measurement. This study was carried out in accordance with the relevant European legislation and Virbac’s chart of Ethics.

A total of one hundred and seventy two individual appetite assessments were made in 5 groups of male and female adult cats to test the palatability of 5 oral supplements. All products were administered 3 hours after the morning meal. Powder, liquid and paste supplements were given with 5 g of kibble to provide a base for the products. The key characteristics (age, gender, weight, normal appetite levels) of all 5 groups were similar on the test day (Table 1). The cats were housed in stable small groups, but were placed in individual boxes for the purposes of the test.

**Table 1:** Animal characteristics in each test group

**Product**	**Number of cats**	**Mean age (years)**	**Mean weight (kg)**	**Sex ratio and sterilisation status**	**Normal appetite level (g/day)**	**Number of feedings per day**	**Usual food**
**Azodyl®**	34	4	3.87	12 females	55 to 70 g	2	Premium or Super premium kibbles (High diversity)
				5 sterilised females			
				17 neutered males			
**Ipakitine®**	35	3.5	3.69	13 females	55 to 70 g	2	Premium or Super premium kibbles (High diversity)
				5 sterilised females			
				17 neutered males			
**Pronefra®**	37	3.5	3.83	13 females	55 to 70 g	2	Premium or Super premium kibbles (High diversity)
				5 sterilised females			
				19 neutered males			
**Renalzin®**	35	3 .5	3.69	13 females	55 to 70 g	2	Premium or Super premium kibbles (High diversity)
				4 sterilised females			
				18 neutered males			
**Rubenal®**	31	4	3.87	9 females	55 to 70 g	2	Premium or Super premium kibbles(High diversity)
				5 sterilised females			
				17 neutered males			

Coupled with thorough statistical analyses, the observations recorded were designed to establish the acceptability and consumption of each supplement. Video records were obtained to allow independent assessment without interfering with the cat’s behaviour. Each animal was offered its allocated test product in its box during 10 minutes. Consumption and prehension were assessed as described below.

Consumption was classified using four categories:

•Ingestion of >95% of the product was defined as total consumption.

•Ingestion of 50-95% of the product was defined as good partial consumption.

•Ingestion of ≥10% but <50% of the product was defined poor partial consumption.

•Ingestion of <10% of the product was defined as a refusal to consume the product.

Ingestion of ≥50% (total plus good partial consumption) was assumed to represent a useful level of consumption. This was therefore defined as useful consumption.

Prehension was defined as the animal voluntarily taking the product in the mouth, whether or not it was then subsequently consumed.

### Tested products

The five tested products (see Table 2 for details) were: Azodyl®, (Vetoquinol, USA), Ipakitine®, (Vetoquinol, France), Pronefra®, (Virbac, France), Renalzin®, (Bayer, France), Rubenal®, (Vetoquinol, France). The tested products were commercially available batches bought through veterinary wholesalers for the four existing products. The new product (Pronefra®) was supplied by the manufacturer. Three products contained IPB (Ipakitine®, Pronefra®, Renalzin®), three products contained intestinal N-binders (Azodyl®, Ipakitine®, Pronefra®), two products contained natural agents thought to contribute to the maintenance of normal kidney architecture (Pronefra®, Rubenal®).

**Table 2:** Product characteristics and administration method used

**Name**	**Content**	**Galenic form**	**Manufacturer’s guidelines**	**Administration method used**
Azodyl	Bacterial products: (Kibow Biotics®: E. thermophilus (KB 19), L. acidophilus (KB 27), B. longum (KB 31))	Powder in single dose capsules	1 to 3 capsules daily depending on weight	One capsule of powder spread over 5 g of kibble
	Psyllium husk			
Ipakitine	-IPB (calcium carbonate)	Powder	One spoon of powder (1 g) per 5 kg of BW, spread on the food	One spoon of powder spread on 5 g of kibble
	-Chitosan			
Pronefra	-IPB (calcium carbonate and magnesium carbonate)	Liquid suspension	0.25 ml/kg twice daily with food	2 ml spread on 5 g of kibble
	-Chitosan			
	-Astragalus membranaceus			
	-Hydrolysate of fish protein			
Renalzin	-IPB (lanthanum carbonate)	Paste	Two presses (~2 ml) per cat, spread on the food	One press of paste spread on 5 g of kibble
Rubena75	-Rheum officinale, extract	Tablet	2 tablets per cat daily	1 tablet placed in the bowl

### Statistical analysis

Statistical analyses were performed using SAS 9.3. The parameters prehension, total consumption and useful consumption (as defined above) were compared between groups using a binomial model with group as fixed factor.

In case of significant group effect, pair-wise comparisons were performed between each pair of groups (Likelihood ratio or Wald chi square test).

The significant threshold was set at 5%.

Each product was therefore compared to each other product for the following parameters:

•Prehension

•Total consumption

•Useful consumption (total plus good partial consumption).

## Results

**Figure 1:**
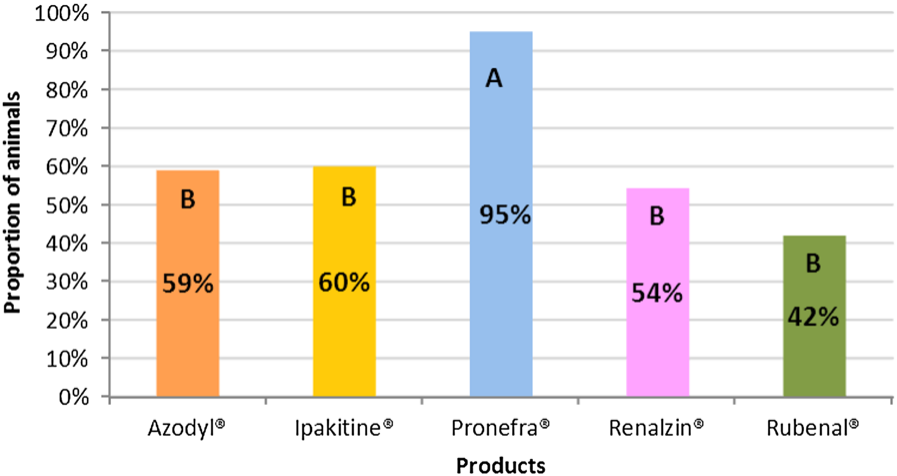
**Prehension of CKD supplements in cats.** This chart presents the level of spontaneous prehension for the tested products and demonstrates two statistically different groups, A and B. Columns not bearing a similar letter are significantly different.

The percentage of prehension noted in each group is displayed in Figure 1. When statistical analysis of the prehension rate was performed, two distinct groups were noted. Group A was composed of Pronefra®, and group B was composed of Azodyl®, Ipakitine®, Renalzin® and Rubenal®. Spontaneous prehension of the only product in group A (Pronefra®) was found to be statistically superior to each of the products in group B. No statistical difference in spontaneous prehension was found between any of the products in group B. See Table 3 and Figure 1 for details.

**Table 3:** P-values for the pairwise comparisons of the level of prehension, total consumption, useful consumption and refusal for each product

**Product comparisons**	**Prehension**	**Total consumption**	**Useful consumption**	**Refusal**
**Ipakitine vs Renalzin**	0.6293	1.0000	0.0005*	0.3383
**Ipakitine vs Azodyl**	0.9207	0.0011*	<.0001*	0.7038
**Ipakitine vs Rubenal**	0.1450	0.0786	0.0118*	0.0076
**Ipakitine vs Pronefra**	0.0023*	<.0001*	<.0001*	<.0001*
**Renalzin vs Azodil**	0.7039	0.0011*	0.0336*	0.1838
**Renalzin vs Rubenal**	0.3176	0.0786	0.2906	0.0784
**Renalzin vs Pronefra**	0.0008*	<.0001*	<.0001*	<.0001*
**Azodyl vs Rubenal**	0.1758	0.0900	0.0022*	0.0026*
**Azodyl vs Pronefra**	0.0019*	0.1088	0.0459*	0.0002*
**Rubenal vs Pronefra**	<.0001*	0.0013*	<.0001*	<.0001*

Where products are significantly different, this is noted in bold with an asterisk.

**Figure 2:**
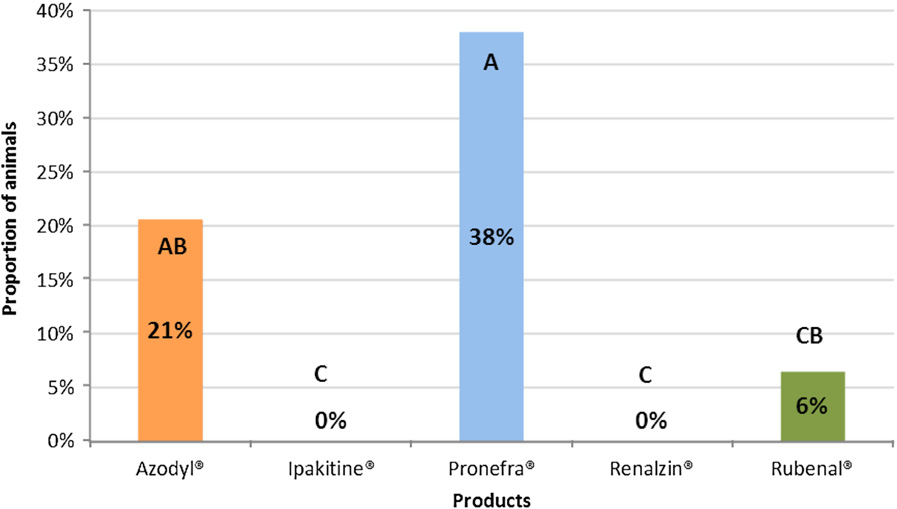
**Total consumption of CKD supplements in cats.** The bars indicate the percentage of cats which consumed more than 95% of the product in each group. Columns not bearing a similar letter are significantly different.

Total consumption data is displayed in Figure 2. When statistical analysis of total consumption was performed, four distinct groups were noted. The groups were named such that groups which were statistically different did not contain a common letter. Specifically: Group A was composed of Pronefra®, group AB was composed of Azodyl®, group CB was composed of Rubenal®, and group C was composed of Ipakitine® and Renalzin® The level of total consumption was found to be statistically superior for statistical group A when compared to groups CB and C. Group AB was also superior to group C. No statistical difference was found between groups A and AB, between groups AB and BC, or between groups BC and C. See Table 3 and Figure 2 for details.

**Figure 3:**
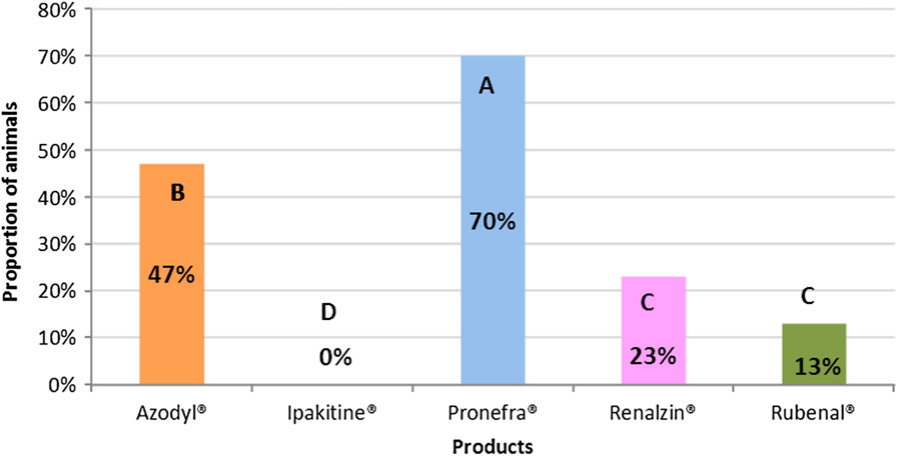
**Useful consumption of CKD supplements in cats.** The bars indicate the percentage of cats which consumed more than 50% of the product in each group. Columns not bearing a similar letter are significantly different.

**Figure 4:**
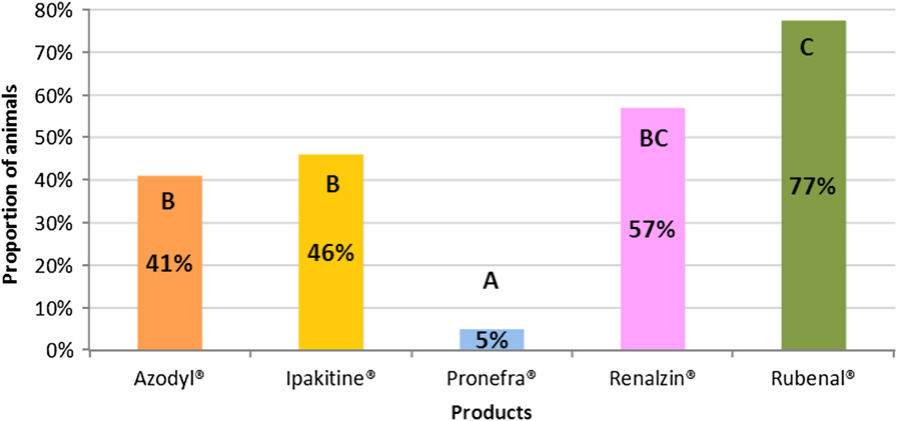
**Refusal to consume CKD supplements in cats.** The bars indicate the percentage of cats which consumed less than 10% of the product in each group. Columns not bearing a similar letter are significantly different.

Useful consumption data (defined as ingestion of ≥50%) are presented in Figure 3. When statistical analysis of useful consumption was performed, four distinct groups were noted. Group A was composed of Pronefra®, group B was composed of Azodyl®, group C was composed of Rubenal® and Renalzin® and group D was composed of Ipakitine®. The level of useful consumption was found to be statistically superior for statistical group A (product Pronefra®) when compared to all other groups (B, C and D). Group D (product Ipakitine®) was found to be statistically inferior to all other groups (A, B and C) (see Table 3 and Figure 3 for details). Data relating to refusal to consume supplements is presented in Table 3 and Figure 4.

## Discussion

One objective of this study design was to model the situation of cats in the early stages of the disease or cats with strong food preferences where IPB are added to food with a normal protein content. In fact, as the disease progresses in cats they become more and more anorexic [4] and unwilling to accept new flavours. This means that it is desirable to introduce any supplements at an early stage after diagnosis of CKD, ideally without any deleterious effect on the global food consumption. This is particularly the case for IPBs, where it has been shown that they can be efficient even before obvious hyperphosphatemia is identified [11,12,19,21].

It is possible that healthy cats are not well representative of the differences between product palatabilty in sick cats. This is a major limitation of the study. However, use of healthy cats avoids the large variability in appetite levels seen in sick cats, avoids the ethical problems seen when testing products in cats which are already stabilised on appropriate management options and avoids the risks of interference due to previous exposure to the products. It could be interesting to attempt to follow the products in the field as a prospective study, but this would require very large numbers of cats to provide meaningful results.

For the four existing presentations (Azodyl®, Ipakitine®, Renalzin® and Rubenal®) prehension was shown to be rather low in healthy cats. Voluntary prehension is not always easy to achieve in cats, but is clearly the first requirement in cases where the owner is unwilling or unable to use a forced administration for the animal. Tablet presentations are often more adapted to forced administration unless they are highly palatable.

For the total consumption data, the statistical analysis revealed more complexity with four overlapping groups (Figure 2 and Table 3); despite this complexity the most clear contrast revealed was within the products containing IPB. As noted previously, the most important goal for nutritional management of cats with CKD is the reduction of serum phosphorus which has been shown to correlate with extended lifespan [10-15]. We did not see extremely high proportions of cats willing to consume the product totally for any product containing IPB. However, the fact that no cats were willing to totally consume the product for the two IPB-containing supplements in group C (Ipakitine® and Renalzin®) is potentially a concern. The significantly higher result for Pronefra® may represent an important benefit in the compliance of cats requiring IPB administration. As seen with prehension data, no statistical difference was noted between the two products not containing IPB.

Cats are well known to be a species inclined to neophobia [24], and more specifically cats with CKD are known to have problems with their appetite [4]. Thus to obtain 100% consumption of a newly introduced product is difficult and a consumption rate that exceeds 50-70% of the product could be a desirable target. It could also be reasonably argued that at least 50% of the recommended amount may be assumed to be necessary to provide obvious benefit for the animal. For this reason we defined consumption as useful when more than 50% of the delivered product was willingly consumed by the cat. In contrast, consumption not exceeding 10% was defined as refusal given the fact that it is unreasonable to expect any real benefit from a product consumed at this rate.

The useful consumption statistical analyses once again demonstrated the existence of a distinct difference among the products containing IPB. Once again Pronefra® stood out as being distinctly separated from Renalzin® and Ipakitine®. However in this analysis around one quarter of the cats were willing to consume amounts of Renalzin® that were defined as useful. Once again, and somewhat surprisingly, no cats were willing to consume useful amounts of Ipakitine®. This low innate palatability combined with its galenic presentation as a powder which makes forced administration extremely difficult will almost certainly compromise compliance. For the analysis of useful consumption, the situation for the products not containing an IPB was different to that for total consumption and prehension: Azodyl® was found to be statistically superior to Rubenal® and was consumed to a useful level by around half of the cats.

The difference between products containing an IPB appears to be even more pronounced when refusal to consume is assessed with around half of all cats refusing to consume the two previously existing products.

The products in this study were administered with dry food. It is possible that use of canned foods may have produced a different result due to the inherent palatability of the wet food which may be used to mask to low palatability of the supplements. However the purpose of this study was to assess the inherent palatability of the supplements in isolation from any effect of the food. It is obvious that consumption of less than 50% of the delivered product (and especially for products containing IPB) would be a critical limitation and seriously compromise the final goal of the supplementation if this was sustained over the longer term.

Additionally it could be emphasised from the data obtained in this study that in cases where a practitioner wishes to supplement a feline patient with not only IPB, but also with a uremic toxin binder and eventually with a supplement to help maintain normal kidney architecture with the presentations which are currently available on the market he/she will face the necessity to administer three different formulations each with a rather low inherent palatability. This low palatability may compromise compliance with supplement use in patients and owners and may help explain the very poor market penetration of CKD supplements [22,23]. For the products containing IPB, the lack of palatability could be easily explained by the nature of IPB, which are known to have a lack of smell and taste: mostly they are simple molecular entities or polymeric structures able to form insoluble compounds with digestible phosphorus. Lack of smell could be easily justified to human patients by medical necessity, but it is not the case for animals. Thus, for the manufacturers of supplements designed for CKD animals and especially for cats, palatability should be one of the highest priorities unless the product is designed only for use when mixed with highly palatable food.

The best performing product of the four currently on the market was Azodyl®. One key point to note about this study was that the capsules of Azodyl® were opened and the contents applied to the food, while the manufacturer’s recommendation is to administer it as an entire capsule. It is extremely rare for cats to voluntarily consume a capsule without any forced administration. However, although not all owners are able or willing to forcibly administer products to their cats, for those who are experienced, direct administration of a capsule guarantees that the entire dose is received by the animal. It is probable that the technique we used is relatively widespread in the field.

The results of this study suggest that the galenic form of the supplements could be an important factor. Rubenal® is presented as a tablet, where, once again, if the owner is experienced enough, forced administration could ensure that the complete dose was received on each occasion. However not all cats are amenable to this. The refusal of the product by the majority of the cats may be linked to the absence of a specific palatability agent and the large size of the tablet (approximately 15 mm). It could have been possible to try to crush the tablet to form a powder, and it may have been interesting to have observed whether this may have reduced the proportion of cats which totally refused to consume the product. Likewise, the co-administration with highly palatable food may have had an impact on the results with this product.

It could be speculated that in some cases even food ingestion could be compromised if unpalatable supplements are added to the food, given the specificity of cats regarding feeding [24]. Additionally, some reports have shown a liquid form, compared to tablets and capsules, could be more efficient for intestinal binders [5] without taking into account the amount of the supplement ingested. Finally, given the frequent appetite problems in CKD animals and especially in cats, supplements for renal disease should allow the owner, in cases of necessity, to force the administration. Anorexia in cats with chronic kidney disease is often related to the intensity of uraemia and acidosis [5]. Administration of medication and appropriate supplements regularly may allow decreased absorption of dietary phosphorus, thus preventing or decreasing hyperphosphatemia and secondary hyperparathyroidism, and may even eventually attenuate uraemia [6] and related clinical signs to help avoid malnutrition and weight loss in feline patients. In this case if the palatability of the product is acceptable, the animal may be willing to ingest it by itself thus avoiding life-time forced administration and facilitating ease of use for the owner. However having a palatable presentation in a form that also allows forced administration where necessary could be the ideal situation. In the present study the only supplement shown to have an acceptable level of palatability was Pronefra®.

## Conclusion

One product in this study (Pronefra®) was clearly demonstrated to have a higher prehension and consumption rate. This could potentially help change the image of feline CKD supplements which are perceived as products with low palatability and instead allow the practitioner to propose early nutritional intervention with multiple benefits and a high chance of spontaneous intake in feline patients suffering from chronic renal disease.

## Competing interests

Authors NB, SF, DM, PM and HG are employees of Virbac. Virbac paid for this research which relates to a potential future product in development.

## Authors’ contributions

PM, HG conceived the study. NB, SF, DM were responsible for analysis and interpretation of the data. NB, DM drafted the paper. All authors contributed to critical revision of the paper and approved the final version.
